# Flash VEP-P2 latency delay in Alzheimer’s disease and cognitive impairment: a systematic review and meta-analysis

**DOI:** 10.3389/fneur.2026.1772949

**Published:** 2026-08-10

**Authors:** Sumeng Song, Yajuan Li, Liangdong Lin, Lei Duan

**Affiliations:** 1 Department of Neurology, Huai’an No. 3 People’s Hospital, Huai’an, China; 2 Huai’an Second Clinical College of Xuzhou Medical University, Huai’an, China

**Keywords:** Alzheimer’s disease, biomarker, dementia, flash visual evoked potential P2 component, meta-analysis

## Abstract

**Background:**

The flash visual evoked potential P2 component (FVEP-P2) has been investigated as a potential neurophysiological measure of cognitive impairment, but its biological and clinical significance remains uncertain. However, evidence remains fragmented. This systematic review and meta-analysis aimed to quantitatively synthesize evidence on FVEP-P2 latency alterations across the cognitive impairment continuum, including mild cognitive impairment (MCI) and major neurocognitive disorders (dementia).

**Methods:**

We systematically searched PubMed, Embase, Web of Science, and Cochrane Library until July 28, 2025. Standardized mean differences (SMDs) for FVEP-P2 latency were pooled from 21 studies (*n* = 1,778) using a random-effects model to compare AD, mild cognitive impairment (MCI), and VaD groups against healthy controls (HC). Subgroup and sensitivity analyses explored heterogeneity sources.

**Results:**

Twenty-one studies involving 1,778 participants were included. FVEP-P2 latency was significantly prolonged in patients with AD compared with healthy controls (18 comparisons, SMD = 1.55, 95% CI 1.05–2.05, I^2^ = 88.9%). Exploratory analyses also suggested prolonged latency in VaD (four comparisons; SMD = 1.44, 95% CI 0.35–2.53; I^2^ = 92.2%), MCI (two comparisons; SMD = 1.50, 95% CI 0.26–2.74; I^2^ = 53.8%), and PDD (one comparison; SMD = 1.44, 95% CI 0.63–2.25). In a restricted exploratory analysis excluding six selected AD comparisons, the association remained statistically significant and directionally consistent (SMD = 1.32, 95% CI 0.83–1.82; I^2^ = 84%). The ≥ 75-year subgroup showed lower heterogeneity (I^2^ = 41%), although differences between age subgroups were not statistically significant (*p* = 0.56). Funnel plot asymmetry and Egger’s test suggested potential small-study effects in the AD analysis.

**Conclusion:**

Prolonged FVEP-P2 latency was observed in AD, with preliminary findings suggesting similar alterations in other forms or stages of cognitive impairment. These findings do not establish a specific neurobiological mechanism or diagnostic role. Further studies are needed to clarify the biological basis and clinical value of FVEP-P2 latency.

## Introduction

Neurodegenerative diseases, particularly dementia, pose a major global public health challenge. In China, a large-scale national cross-sectional study (n = 46,011 adults aged ≥60 years) estimated the overall prevalence of dementia at 6.0%, with Alzheimer’s disease (AD) representing the predominant subtype (3.9%), followed by vascular dementia (VaD, also referred to as vascular neurocognitive disorder in DSM-5; 1.6%) and other types (0.5%); mild cognitive impairment (MCI)—a high-risk prodromal stage of dementia—exhibited a prevalence of 15.5% in the same population (1, 2). Globally, over 57 million people were living with dementia in 2021, with Alzheimer’s disease accounting for 60–70% of all cases, and the number is projected to reach 139 million by 2050 (3). This high prevalence not only drives progressive cognitive decline and loss of self-care capacity, but also imposes substantial socioeconomic and psychological burdens on patients and families.

Flash visual evoked potential (FVEP) is a non-invasive and technically accessible electrophysiological technique. Its P2 component is thought to involve activity within visual and higher-order association areas and may provide information on cortical visual processing, although its precise physiological determinants remain incompletely understood (4). In neurodegenerative disorders including AD, VaD, and dementia with Lewy bodies (DLB, or neurocognitive disorder with Lewy bodies), FVEP-P2 demonstrates prolonged latency or reduced amplitude (5, 6). Prolonged FVEP-P2 latency has been reported in several cognitive disorders, but its neurobiological basis remains uncertain. It may be influenced by alterations in visual pathway function, cortical processing, neurotransmission, or other disease-related factors (4, 7). Consequently, FVEP-P2 has been investigated as a potential adjunctive neurophysiological marker in the assessment of cognitive impairment (1, 8). Recent years have witnessed growing research on FVEP-P2’s diagnostic value across dementia subtypes (e.g., AD, VaD). However, current evidence derives predominantly from single-center observational studies with limited sample sizes, exhibiting significant methodological heterogeneity (5). No consensus exists regarding subtype-specific variations in FVEP-P2 signatures. Evidence for individual subtypes remains limited, and conclusions are inconsistent (9). Although AD, VaD, MCI, and PDD differ substantially in their primary etiologies and pathological mechanisms, they may converge on downstream disturbances in cortical visual-information processing. FVEP-P2, which reflects activity within visual and higher-order association networks, may therefore capture a non-specific electrophysiological consequence of impaired cortical signal processing rather than a disease-specific pathological mechanism. This provides a physiological rationale for comparing the direction and magnitude of FVEP-P2 latency alterations across different forms and stages of cognitive impairment. Importantly, synthesis within this electrophysiological framework does not imply shared etiology or mechanistic equivalence among these conditions; accordingly, subtype-specific analyses were regarded as the principal findings, whereas the overall cross-etiology estimate was considered exploratory. This systematic review and meta-analysis aimed to quantify FVEP-P2 latency differences between patients with AD, VaD, PDD, or MCI and healthy controls, and to examine the consistency of these differences across clinical populations.

## Methods

### Literature retrieval

This systematic review adheres to the Preferred Reporting Items for Systematic Reviews and Meta-Analyses (PRISMA) guidelines (10), and retrospectively registered on PROSPERO (CRD420261389240). Completed checks confirm that all PRISMA items are addressed in the manuscript. We systematically searched four databases: PubMed,^1^ Embase,^2^ Web of Science,^3^ and the Cochrane Library,^4^ with the search conducted from inception to July 28, 2025. No lower date limit was applied because the available evidence on FVEP-P2 latency in dementia is limited and includes many studies published during the early development of this technique. Two independent researchers performed the systematic search for relevant studies using a combination of Medical Subject Headings (MeSH) and free-text terms related to “dementia” and “flash visual evoked potential,” along with their synonyms. The complete search strings for each database are provided in Supplementary material. During the initial search process, we did not impose any restrictions on language or study design to ensure broad coverage. However, the final inclusion criteria were restricted to English-language publications due to resource constraints for accurate translation and independent data verification of non-English studies. This decision may have introduced language bias and potentially excluded relevant studies published in other languages; this limitation is discussed further in the Discussion section. Data extraction was independently completed by two researchers: one performed the data extraction using a standardized table, and the other reviewed the extracted data. Any discrepancies were resolved through discussion until a consensus was reached. The complete and detailed search strings for each database are provided in Supplementary materials-Search Strategy.

### Inclusion and exclusion criteria

Inclusion criteria: (1) original comparative studies evaluating FVEP-P2 latency in participants with dementia or MCI and a healthy control group were eligible. Eligible designs included case–control studies, cohort studies, and diagnostic-accuracy studies; (2) participants clinically diagnosed with major neurocognitive disorder (dementia; e.g., AD, FTD, DLB, PDD) or mild cognitive impairment (MCI), with an established healthy control group (HC); MCI was included as a prodromal cognitive impairment stage given its established clinical and neurophysiological overlap with early dementia; (3) reported FVEP-P2 latency as mean ± standard deviation (SD), or as median with interquartile range (IQR) convertible to mean ± SD using established formulas from the Cochrane Handbook; (4) Original English-language publications (reviews, meta-analyses, conference abstracts, and editorials excluded). While the initial database search was conducted without language restrictions to maximize recall, only English-language studies were retained in the final analysis due to resource constraints for accurate translation and independent data verification.

Exclusion criteria: (1) conference abstracts, editorials, case reports (samples <2 subjects), reviews, meta-analyses, preclinical/animal studies; (2) studies with Newcastle-Ottawa Scale (NOS) score < 5 were excluded according to the minimum methodological-quality criterion applied during full-text eligibility assessment; (3) patients with severe ocular diseases (e.g., glaucoma, macular degeneration) or central nervous system lesions affecting visual pathways; (4) duplicate publications (retaining the most recent version). Two reviewers independently screened studies. Discrepancies were resolved through consensus discussion with a third reviewer; (5) for diagnostic accuracy studies originating from the same research group or institution, participant characteristics (including sample size, recruitment period, and clinical setting) were cross-checked to identify potential patient overlap; where overlap was confirmed or strongly suspected, only the study with the larger sample size or more complete data was retained; (6) studies that did not report FVEP-P2 latency as a primary outcome (e.g., reporting only P100 latency or VEP amplitude; *n* = 7), or studies in which outcome data were presented exclusively in graphical format without extractable mean ± SD values (*n* = 4).

### Risk of bias assessment

The methodological quality of the included studies was evaluated using the NOS and QUADAS-C (Quality Assessment of Diagnostic Accuracy Studies—Comparative) tools. The NOS is a validated tool for assessing the quality of case–control and cohort studies in meta-analyses, employing a star system across three domains: selection, comparability, and outcome/exposure (11). QUADAS-C is a validated tool specifically designed for evaluating diagnostic meta-analyses (12). This tool examines four areas: patient selection, indicator testing, reference standards, and process and scheduling. The bias risk of each domain was classified as high, low, or unclear based on predefined criteria using Review Manager 5.4. Two investigators independently conducted quality assessments and resolved differences through discussions with a third reviewer to reach a consensus.

### Data extraction and outcome definition

Two reviewers independently extracted data using a standardized data-extraction form. The extracted information included the first author, publication year, country, study design, disease subtype, diagnostic criteria, sample size, participant age, cognitive assessment scores, FVEP equipment, stimulation parameters, electrode configuration, P2-identification method, and FVEP-P2 latency in the patient and healthy control groups. Disagreements were resolved through discussion, with consultation from a third reviewer when necessary. FVEP-P2 latency was the outcome of interest and was extracted separately for each eligible disease subgroup. FVEP-P2 was identified according to the component designated as P2 by the original study, generally corresponding to the second positive deflection following flash stimulation. Peaks were not reclassified solely on the basis of their latency. Continuous data were preferentially extracted as mean ± standard deviation (SD). When results were reported as medians with interquartile ranges or other convertible summary statistics, means and SDs were estimated using established methods recommended in the Cochrane Handbook (13). If a study reported more than one eligible dementia subtype, data for each subtype were extracted separately for the corresponding disease-specific analysis.

### Statistical analysis

Statistical analyses were performed using Review Manager version 5.4 (The Cochrane Collaboration, Copenhagen, Denmark) and R version 4.4.3 with the metafor package. Review Manager 5.4 was used to generate forest plots and for visual inspection of the funnel plot, whereas R was used for the meta-regression analyses and Egger’s regression test. The standardized mean difference (SMD) with 95% confidence interval (CI) was used to summarize differences in FVEP-P2 latency between patient and healthy control groups because the included studies used heterogeneous FVEP equipment and stimulation protocols. Effect sizes were pooled using inverse-variance-weighted random-effects models. In addition to 95% confidence intervals, 95% prediction intervals were reported for disease-specific pooled analyses containing at least two comparisons to describe the range of effects that might be expected in a future study. These analyses synthesized continuous between-group differences in FVEP-P2 latency rather than diagnostic accuracy measures such as sensitivity, specificity, or area under the receiver operating characteristic curve; therefore, the pooled SMDs reflect group-level separation and do not establish diagnostic thresholds or screening performance.

Statistical heterogeneity was assessed using Cochran’s Q test and the I^2^ statistic, with *p* < 0.10 for the Q test indicating statistically detectable heterogeneity. I^2^ values >50 and >75% were considered to indicate substantial and considerable heterogeneity, respectively. For the AD analysis, an exploratory subgroup analysis was conducted according to mean participant age (< 70, 70–75, and ≥ 75 years). Exploratory univariable meta-regression analyses were performed to examine mean age, mean Mini-Mental State Examination (MMSE) score, publication year, and study design as potential moderators of effect size. Flash intensity was not included as a moderator because it was reported using non-comparable units or device-specific settings across studies, precluding reliable conversion to a common quantitative scale. Given the limited number of studies and the use of aggregate study-level covariates, all subgroup and meta-regression analyses were considered exploratory.

A restricted exploratory sensitivity analysis was conducted for the AD comparison by excluding six selected comparisons and recalculating the pooled effect estimate and heterogeneity. This analysis examined whether the direction and statistical significance of the AD association were sensitive to the inclusion of these comparisons. Potential small-study effects were assessed for the AD analysis, which included 18 comparisons, through visual inspection of the funnel plot and Egger’s regression test; a two-sided *p* < 0.05 for Egger’s test was considered to indicate funnel plot asymmetry.

Although the included studies comprised case–control, cohort, and diagnostic-accuracy designs, all contributed extractable group-level FVEP-P2 latency data for patients and healthy controls, allowing SMDs to be used as a common effect measure. When an individual study reported more than one eligible dementia subtype, each subtype was included as a separate disease-specific comparison. An exploratory overall analysis was also presented across all dementia subtypes. Because some disease-specific comparisons derived from the same study shared a healthy control group, the comparisons may not have been fully independent. Therefore, subtype-specific estimates were regarded as the principal findings, whereas the overall dementia estimate was interpreted only as an exploratory summary. All statistical tests were two-sided, and *p* < 0.05 was considered statistically significant unless otherwise specified.

## Results

### Study selection and characteristics

**Figure 1:**
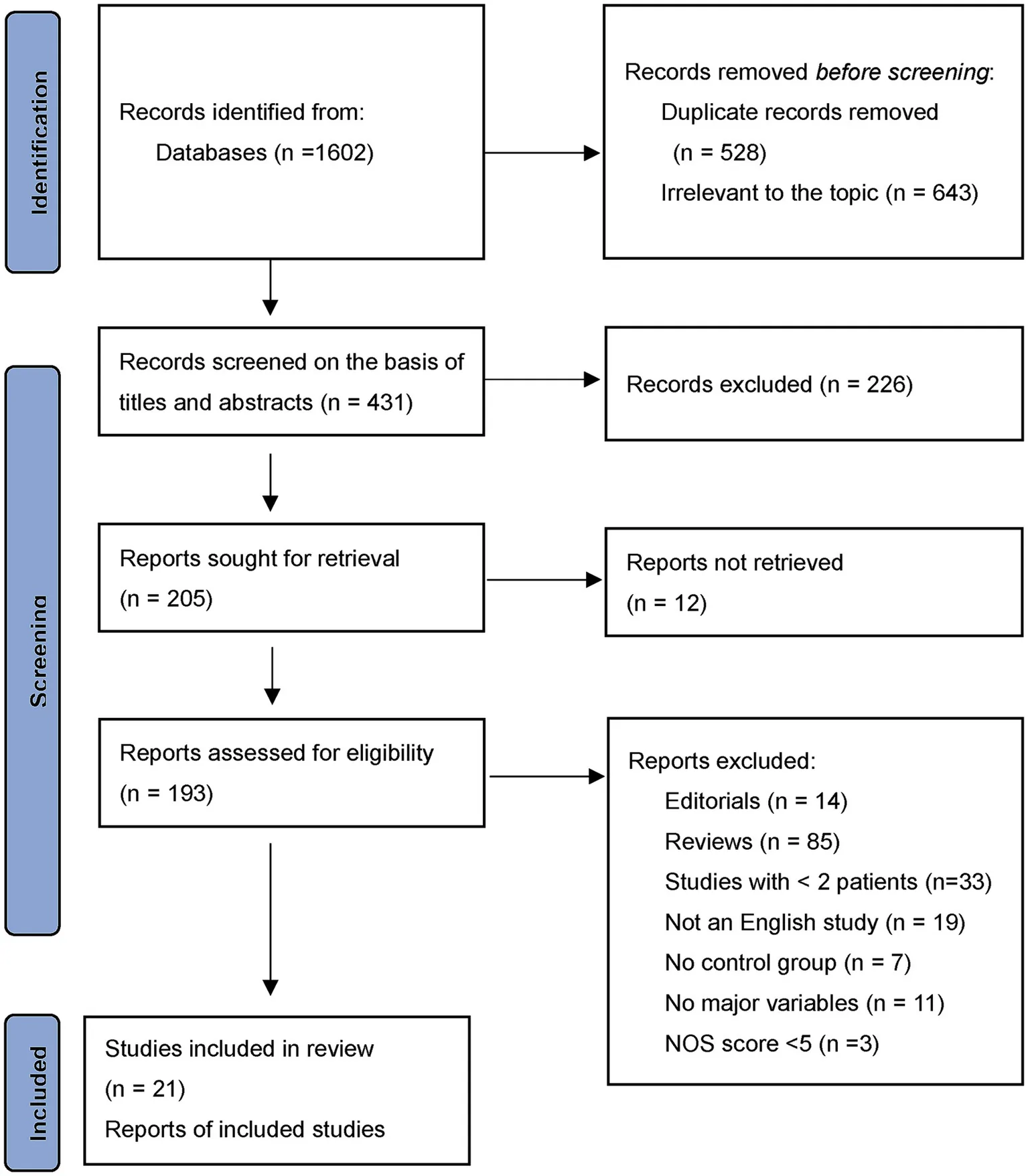
Literature screening flow and results. The number of records retrieved from each database: PubMed (*n* = 1,014), Cochrane Library (*n* = 13), EMbase (*n* = 316), Web of Science (*n* = 259).

The database searches identified 1,602 records from PubMed, Embase, Web of Science, and the Cochrane Library. After the removal of 528 duplicate records and 643 irrelevant records, 431 records underwent title and abstract screening, of which 226 were excluded. Among the 205 reports sought for retrieval, 12 could not be retrieved. The remaining 193 full-text reports were assessed for eligibility, and 172 were excluded for the reasons summarized in Figure 1 and Supplementary Table S1. Ultimately, 21 studies were included in the systematic review and meta-analysis (7, 14–33) (Figure 1). These studies, published between 1983 and 2023, comprised a total sample of 1,778 participants (1,013 patients and 765 controls). The studies covered AD (18 studies), MCI (2 studies), VaD (4 studies), and PDD (1 study) (Table 1). Some studies contained data from multiple disease subgroups. The studies originated from 10 countries, including the United States (6 studies), the United Kingdom (7 studies), and Italy (2 studies). Of these, 12 studies (57.14%) employed a case–control design, while 8 studies (38.10%) were diagnostic accuracy studies and one cohort study (4.76%). FVEP acquisition protocols varied considerably across studies. Flash intensity was reported in several non-comparable units, including joules, candela-based measures, lumen·seconds/m^2^, and device-specific intensity settings, and was not reported in some studies. Among studies reporting a fixed stimulation frequency, values generally ranged from 0.9 to 2 Hz. Electrode configurations also differed, including recordings from Oz, O1/O2, bipolar occipital derivations, and multichannel 10–20 montages, with reference electrodes placed at Fz, Cz, C3/C4, the mastoids, or the earlobes. Detailed stimulation and recording parameters are presented in Supplementary Table S2. Device types included Grass PS series stimulators (6 studies), LED goggles (3 studies), and custom-built flash devices (11 studies). Cognitive assessment tools included the MMSE, a widely used 30-point instrument for assessing global cognitive function (34) (14 studies); and the Clinical Dementia Rating Scale (CDR; 1 study); the remaining studies did not report cognitive score results. The detailed electrophysiological parameters and stimulation conditions of included studies are shown in Supplementary Table S2.

**Table 1:** Characteristics of included studies on flash visual evoked potential P2 component in neurodegenerative diseases.

Study	Country	Study design	Total sample size	Population description	Quality assessment
Coben, 1983 (15)	USA	Case–control	80	Mild AD vs. HC	NOS: 7/9
Wright, 1984 (16)	UK	Diagnostic accuracy	34	Early-onset AD (presenile dementia) vs. HC	QUADAS-C: Unclear Risk
Harding-1985	UK	Diagnostic accuracy	80	Early-onset AD (primary presenile dementia) vs. HC	QUADAS-C: Unclear Risk
Wright, 1986 (18)	UK	Diagnostic accuracy	70	AD vs. HC	QUADAS-C: Unclear Risk
Wright and Furlong, 1988 (19)	UK	Case–control	54	AD vs. VaD vs. HC	NOS: 8/9
Engedal, 1989 (20)	Norway	Diagnostic accuracy	62	AD vs. VaD vs. HC	QUADAS-C: Unclear Risk
Philpot, 1990 (21)	UK	Diagnostic accuracy	38	AD vs. HC	QUADAS-C: Unclear Risk
Brodie, 1992 (22)	UK	Case–control	14	AD vs. HC	NOS: 7/9
Sloan, 1992 (23)	UK	Cohort	102	AD vs. VaD vs. HC	NOS: 6/9
Coburn, 1993 (24)	USA	Case–control	34	AD vs. HC	NOS: 7/9
O’Mahony, 1993 (25)	Ireland	Case–control	29	PDD vs. HC	NOS: 7/9
Haupt, 1994 (26)	Germany	Case–control	30	AD vs. HC	NOS: 8/9
Sloan, 1994 (27)	UK	Case–control	62	AD vs. VaD vs. HC	NOS: 7/9
Grayson, 1995 (29)	USA	Case–control	20	Probable AD vs. HC	NOS: 6/9
Moore, 1995 (28)	USA	Case–control	62	AD vs. HC	NOS: 7/9
Swanwick, 1996 (30)	Ireland	Diagnostic accuracy	38	AD vs. HC	QUADAS-C: High Risk
Ponomareva, 1998 (31)	Russia	Case–control	40	AD vs. HC	NOS: 8/9
Tartaglione, 2012 (32)	Italy	Case–control	30	AD vs. HC	NOS: 8/9
Fix-2015	USA	Diagnostic accuracy	13	MCI vs. HC	QUADAS-C: High Risk
Arruda, 2023 (14)	Multicenter	Diagnostic accuracy	105	AD vs. HC	QUADAS-C: High Risk
Strigaro, 2022 (7)	Italy	Case–control	38	MCI vs. AD vs. HC	NOS: 6/9

AD, Alzheimer’s Disease; HC, Healthy Controls; MCI, Mild Cognitive Impairment; NOS, Newcastle-Ottawa Scale; PDD, Parkinson’s Disease Dementia; QUADAS-C, Quality Assessment of Diagnostic Accuracy Studies; VaD, Vascular Dementia.

### Literature quality evaluation and publication bias

**Figure 2:**
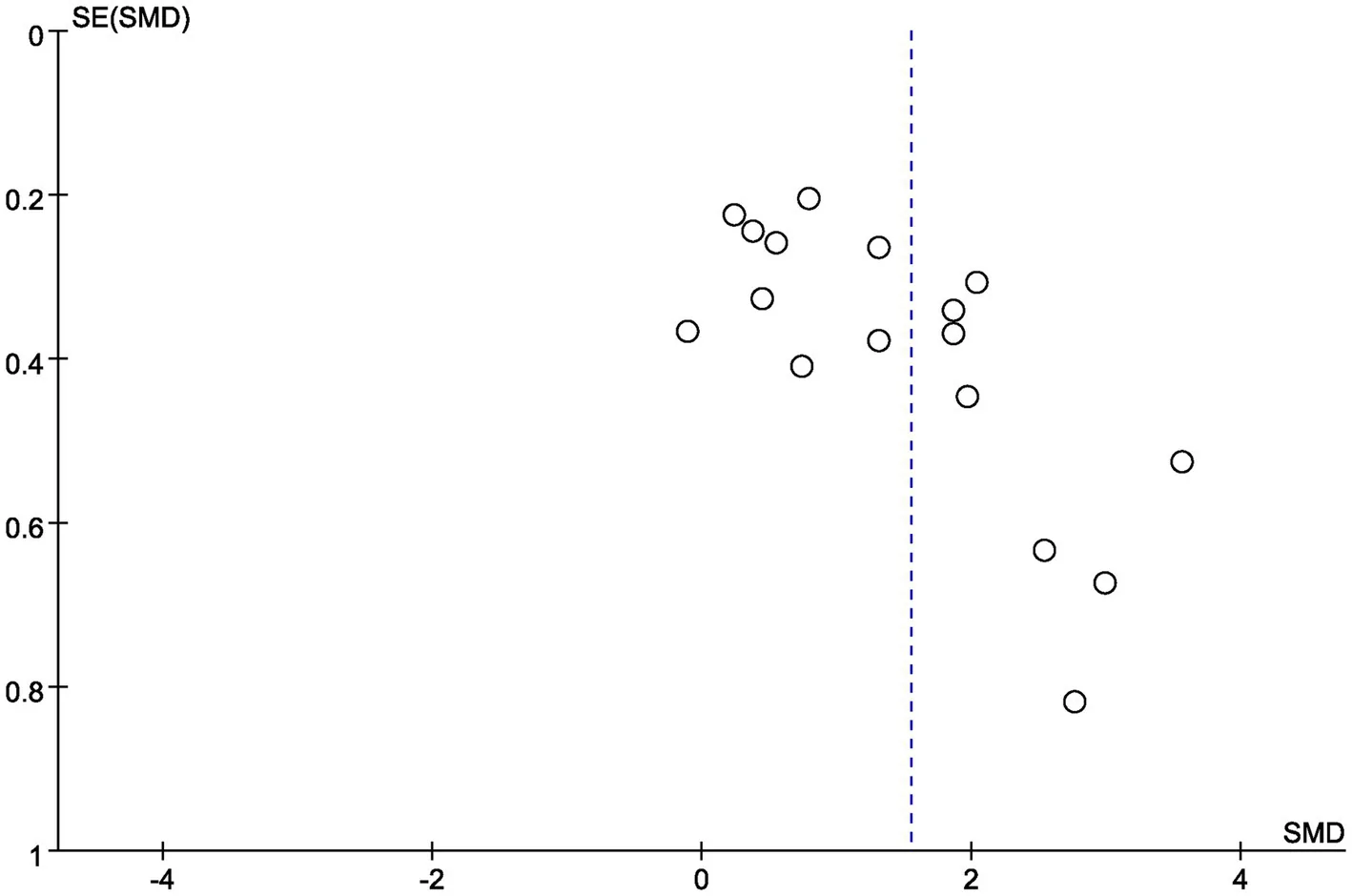
Funnel plot for assessment of potential publication bias (AD vs HC). The funnel plot presents the standard error of the SMD against the SMD for comparisons of FVEP-P2 latency between patients with AD and healthy controls. The asymmetric distribution may indicate small-study effects or selective publication; however, substantial between-study heterogeneity may also have contributed to the observed asymmetry. AD, Alzheimer’s disease; SMD, standardized mean difference.

For the 12 included case–control studies, the NOS scale was used for evaluation. The results showed that the total score ranged from 6 to 8 points (out of 9 points) (Table 2), indicating that the methodological quality was at a medium to good level. The main reason for point loss was the insufficient implementation of blinding in the exposure measurement methods. The NOS score of the cohort study (only one, Sloan-1992) was 6 points, which had limitations in the selection of non-exposed cohorts and the completeness of follow-up results. The diagnostic accuracy studies (8 in total) were evaluated using the QUADAS-C tool. Among the eight diagnostic-accuracy studies, six were judged to have a high risk of bias in the patient-selection domain, primarily because consecutive or random recruitment was not clearly implemented. One study had a high risk of bias in the flow-and-timing domain, and two had a high risk of bias in the reference-standard domain (Tables 3, 4). Due to the limited number of included studies, we conducted a publication bias assessment for the AD subgroup (k = 18). The results of Egger’s linear regression test showed that the statistic for the funnel plot asymmetry test was z = 3.80 and *p* = 0.0001. Visual inspection of the funnel plot (Figure 2) reveals that points representing studies with larger standard errors are predominantly located on the right side of the combined effect size (SMD = 1.55), while there are relatively fewer points on the left, suggesting that small-sample studies with negative or non-significant results may not have been published. Studies with an overall high risk of bias were retained in the primary synthesis. A restricted exploratory sensitivity analysis excluding six selected AD comparisons is reported below (see Sensitivity Analysis section).

**Table 2:** Risk of bias assessment for included case–control studies (stratified analysis).

Study	Selection of study population				Comparability between groups	Measurement of exposure			Total score
	Appropriate case identification	Representativeness of cases	Selection of controls	Definition of controls		Definition of exposure	Ascertainment methods for cases and controls	Non-response rate	
Coben, 1983 (15)	1	1	1	1	2	1	0	0	7
Wright & Furlong, 1988 (19)	1	1	1	1	2	0	1	1	8
Brodie, 1992 (22)	1	0	1	1	1	1	1	1	7
Coburn, 1993 (24)	1	1	1	1	1	1	1	0	7
O’Mahony, 1993 (25)	1	1	1	1	1	1	1	0	7
Haupt, 1994 (26)	1	1	1	1	2	1	0	1	8
Sloan, 1994 (27)	1	1	1	0	2	1	0	1	7
Grayson, 1995 (29)	1	1	1	0	1	1	0	1	6
Moore, 1995 (28)	1	1	1	1	1	1	0	1	7
Ponomareva, 1998 (31)	1	1	1	1	2	1	0	1	8
Tartaglione, 2012 (32)	1	1	1	1	1	1	1	1	8
Strigaro, 2022 (7)	1	1	1	0	1	1	0	1	6

**Table 3:** Risk of bias assessment for included cohort studies (stratified analysis).

Study	Selection of study population				Comparability of cohorts	Outcome measurement			Total score
	Representativeness of exposed cohort	Selection of non-exposed cohort	Ascertainment of exposure	Outcome not present at study start		Outcome assessment	Follow-up adequacy	Follow-up completeness	
Sloan, 1992 (23)	1	0	1	1	2	0	1	0	6

**Table 4:** Risk of bias assessment for included diagnostic accuracy studies.

Included study	Patient selection	Index test	Reference standard	Flow and timing	Overall risk of bias
Wright, 1984 (16)	High risk	Unclear risk	Unclear risk	Low risk	Unclear risk
Harding, 1985 (17)	High risk	Low risk	Low risk	Unclear risk	Unclear risk
Wright, 1986 (18)	High risk	Low risk	Unclear risk	Low risk	Unclear risk
Engedal, 1989 (20)	Low risk	Unclear risk	Low risk	Unclear risk	Unclear risk
Philpot, 1990 (21)	High risk	Unclear risk	Unclear risk	Low risk	Unclear risk
Swanwick, 1996 (30)	High risk	Unclear risk	High risk	Low risk	High risk
Fix, 2015 (33)	High risk	High risk	Unclear risk	Unclear risk	High risk
Arruda, 2023 (14)	Unclear risk	Unclear risk	High risk	High risk	High risk

### Results of meta-analysis

#### FVEP-P2 latency in dementia vs. HC

**Figure 3:**
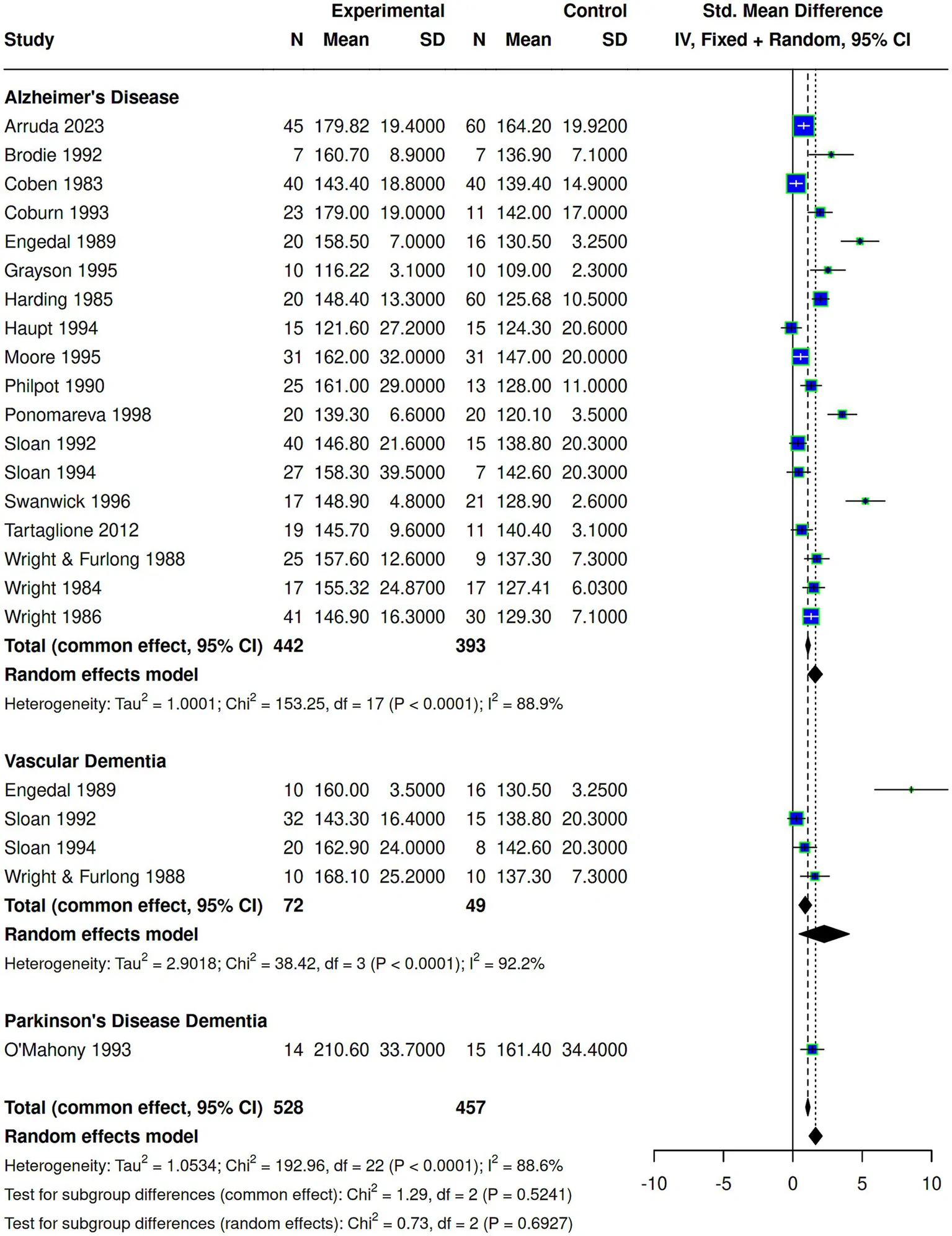
Forest plot of P2 latency in dementia vs. HC. Disease-specific comparisons are presented separately for Alzheimer’s disease (AD), vascular dementia (VaD), and Parkinson’s disease dementia (PDD). Random-effects estimates were used for the principal interpretation. The overall estimate across dementia subtypes should be regarded as exploratory because some disease-specific comparisons derived from the same study shared a healthy control group and were therefore not fully independent. CI, confidence interval; HC, healthy controls; IV, inverse variance; PDD, Parkinson’s disease dementia; SD, standard deviation; SMD, standardized mean difference; VaD, vascular dementia.

We synthesized data from 23 disease-specific comparisons/datasets comparing patients with dementia (due to any etiology) and healthy controls. The overall meta-analysis revealed a significantly prolonged FVEP-P2 latency in the dementia group compared to HC (SMD = 1.64, 95% CI: 1.18 to 2.10, *p* < 0.001; I^2^ = 88.6%). A prediction interval was not reported for the exploratory overall dementia analysis because several comparisons shared a healthy control group and were not fully independent. Subgroup analysis based on dementia etiology showed consistent prolongation across different subtypes: FVEP-P2 latency was significantly prolonged in patients with AD compared with HC (18 comparisons; SMD = 1.55, 95% CI 1.05–2.05, *p* < 0.001; I^2^ = 88.9%). An exploratory analysis of four VaD comparisons also suggested prolonged FVEP-P2 latency, although between-study heterogeneity was considerable (SMD = 1.44, 95% CI 0.35–2.53, *p* = 0.010; I^2^ = 92.2%). The single PDD comparison similarly showed prolonged P2 latency (SMD = 1.44, 95% CI 0.63–2.25, *p* < 0.001) (Figure 3). The 95% prediction interval was −0.46 to 3.55 for the AD analysis and −3.89 to 6.77 for the VaD analysis. A prediction interval was not reported for PDD because only one comparison was available. The VaD prediction interval should be interpreted cautiously because it was based on only four comparisons.

#### FVEP-P2 latency in MCI vs. HC

**Figure 4:**
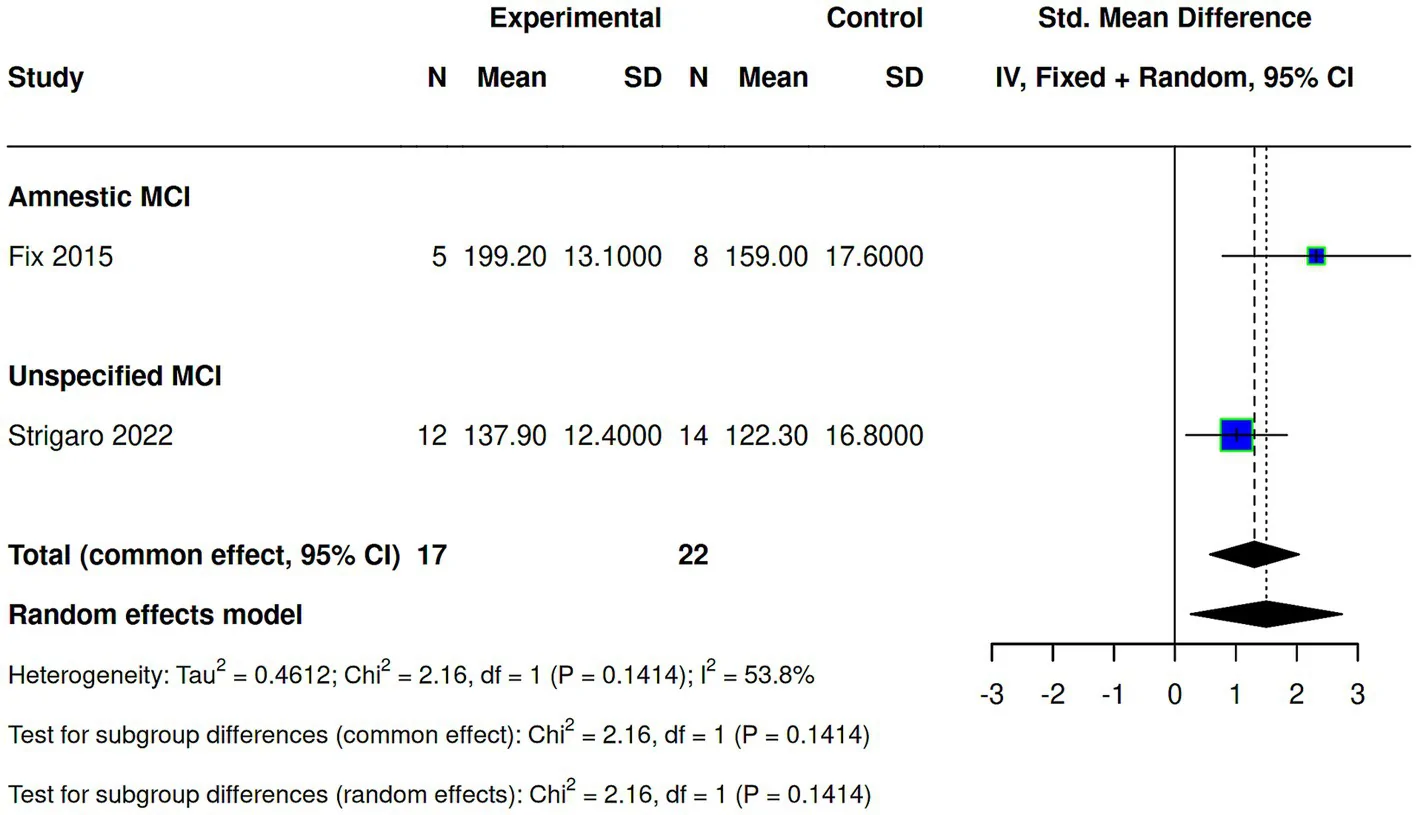
Forest plot of P2 latency in MCI vs. HC. Subgroup analysis was performed based on clinical criteria: Amnestic MCI and Unspecified MCI. CI, confidence interval; HC, healthy controls; IV, inverse variance; I^2^, I-squared; MCI, mild cognitive impairment; SD, standard deviation; SMD, standardized mean difference.

Two studies investigated FVEP-P2 latency in patients with Mild Cognitive Impairment (MCI). To address the etiological heterogeneity of MCI, we performed a subgroup analysis based on the reported clinical criteria. Fix et al. (33) specifically included patients with amnestic MCI (aMCI, highly indicative of Alzheimer’s etiology), while Strigaro et al. (7) included patients with unspecified MCI. The overall pooled analysis showed a significantly prolonged P2 latency in MCI patients compared to HC (SMD = 1.50, 95% CI: 0.26 to 2.74, *p* = 0.018; I^2^ = 53.8%). Both the aMCI subgroup and the unspecified MCI subgroup demonstrated consistent trends of P2 prolongation (Figure 4). However, with only two studies included, this MCI analysis is substantially underpowered and the pooled estimate is highly sensitive to individual study characteristics. These findings should be interpreted strictly as preliminary and hypothesis-generating, necessitating validation through larger, prospective studies. The 95% prediction interval for MCI was −0.32 to 3.31. This interval should be interpreted cautiously because it was based on only two comparisons.

### Sensitivity analysis

**Figure 5:**
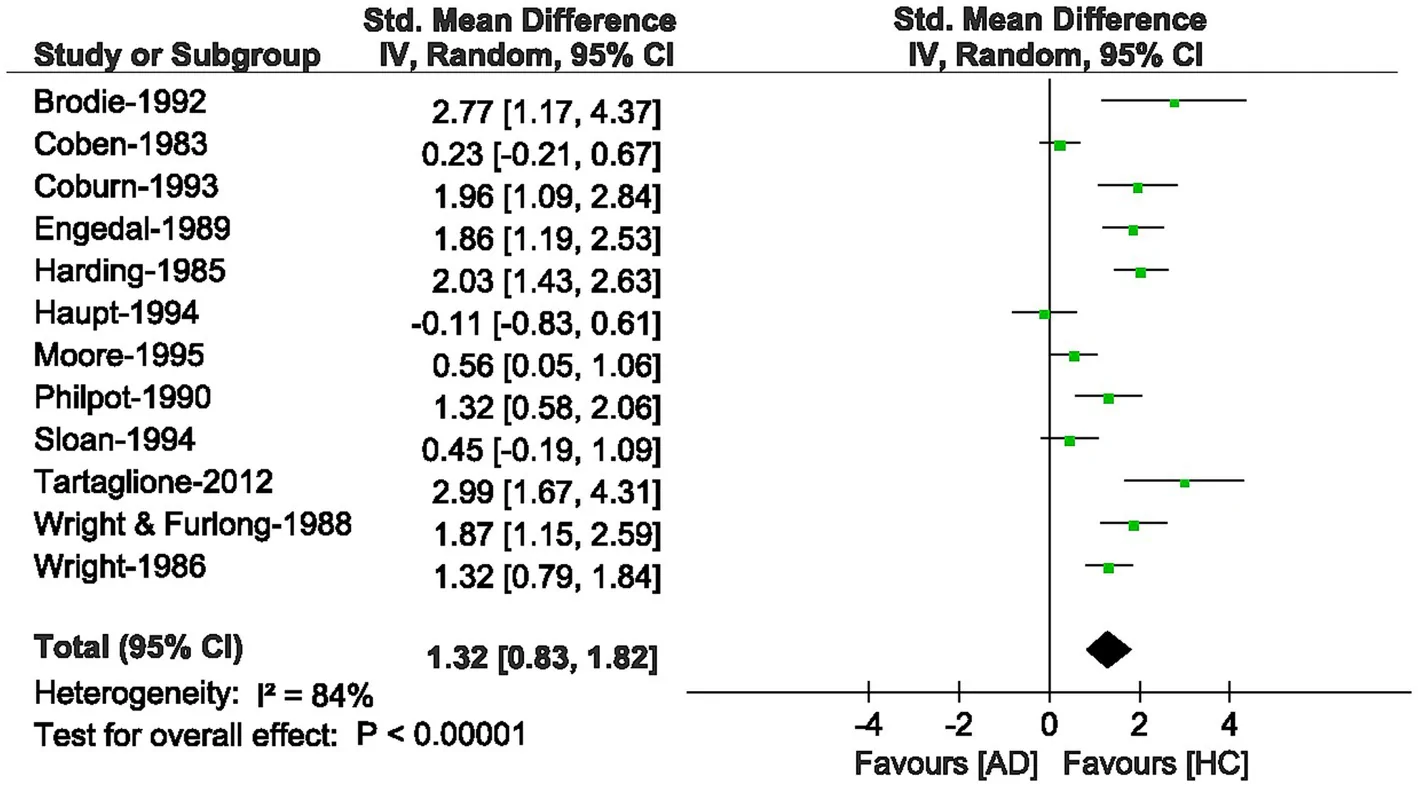
Forest plot of sensitivity analysis. The pooled estimate remained statistically significant and directionally consistent after six selected AD comparisons were excluded, although considerable residual heterogeneity persisted.

A restricted sensitivity analysis was conducted after excluding six AD comparisons (Arruda-2023, Grayson-1995, Ponomareva-1998, Sloan-1992, Swanwick-1996, and Wright-1984). After their exclusion, the association remained statistically significant and directionally consistent (SMD = 1.32, 95% CI 0.83–1.82, *p* < 0.00001), while heterogeneity decreased modestly from 88.9 to 84% (Figure 5). Nevertheless, considerable residual heterogeneity remained. Because this restricted analysis was not based on a uniform quality threshold, it should be interpreted as exploratory. The remaining heterogeneity may reflect variation in diagnostic criteria, disease severity, participant characteristics, ophthalmological screening, stimulation parameters, electrode configurations, peak-identification methods, and recording equipment across studies conducted over approximately four decades.

### Subgroup and meta-regression analyses

**Figure 6:**
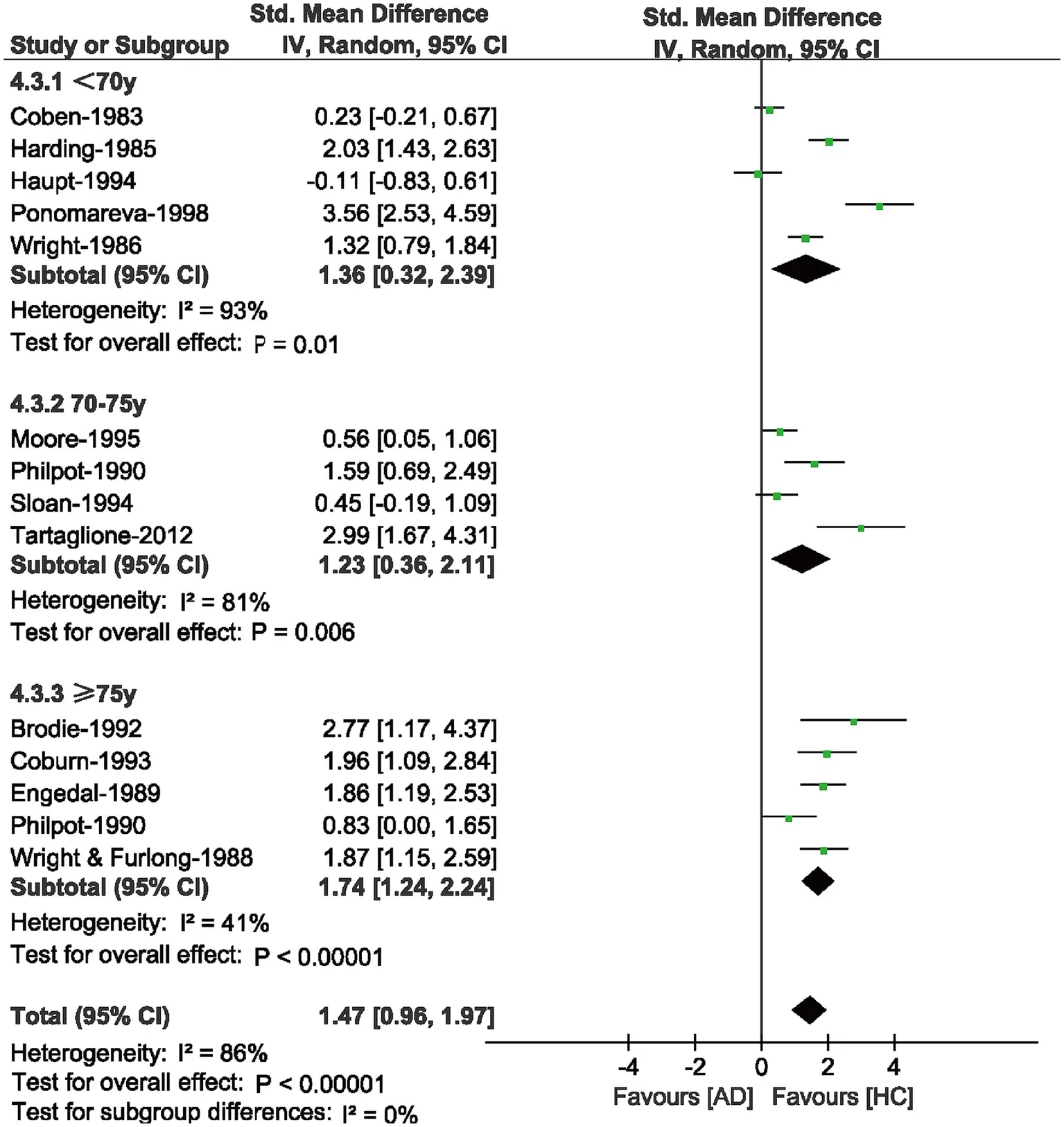
Forest plot of subgroup analysis based on age. CI, Confidence interval; HC, Healthy controls; IV, Inverse variance; I^2^, I-squared; SD, Standard deviation; SMD, Standardized mean difference.

In the exploratory age-stratified analysis, the pooled estimate was SMD = 1.36 (95% CI 0.32–2.39; I^2^ = 93%) for studies with a mean participant age <70 years, SMD = 1.23 (95% CI 0.36–2.11; I^2^ = 81%) for studies with a mean age of 70–75 years, and SMD = 1.74 (95% CI 1.24–2.24; I^2^ = 41%) for studies with a mean age ≥ 75 years (Figure 6). Although the ≥ 75-year subgroup showed lower within-subgroup heterogeneity, the difference among age subgroups was not statistically significant (*p* = 0.56). Therefore, the available data do not provide evidence that mean participant age modifies the association between AD and FVEP-P2 latency.

Study-level differences in diagnostic criteria and P2-identification methods are summarized descriptively in Supplementary Table S3, while reported group-level age data are provided in Supplementary Table S2. The age-stratified findings should be interpreted cautiously because the analyses were based on aggregate study-level mean ages rather than individual-participant data. Moreover, formal age-matching procedures and the comparability of age distributions between patient and control groups were not consistently reported. Residual age-related confounding therefore cannot be excluded. Exploratory univariable meta-regression analyses found no statistically significant associations between effect size and mean age (*p* = 0.491), mean MMSE score (*p* = 0.415), publication year (*p* = 0.602), or study design (*p* = 0.563) (Supplementary Table S4). Although MMSE scores were reported in 14 studies, only nine comparisons provided study-level MMSE data suitable for meta-regression. The non-significant result should therefore not be interpreted as evidence that FVEP-P2 latency is independent of cognitive severity, and this relationship remains inconclusive. Flash intensity could not be examined quantitatively because it was reported using different physical units or device-specific settings that could not be reliably converted to a common scale. Overall, these exploratory analyses should be interpreted cautiously because they were based on aggregate study-level data and included relatively few studies for several covariates.

## Discussion

The diagnostic landscape for neurodegenerative diseases (e.g., AD, VaD) has evolved rapidly, particularly with the advent of blood-based and plasma biomarkers that offer high specificity and reduced invasiveness compared to traditional CSF or PET imaging. Within this modern context, the role of electrophysiological tools must be carefully defined. This systematic review demonstrates significantly prolonged FVEP-P2 latency in AD, with exploratory findings suggesting similar trends in MCI and VaD.

While FVEP-P2 benefits from non-invasiveness, technical simplicity, and low cost, the high heterogeneity and lack of disease specificity indicate that it cannot serve as an independent diagnostic or screening tool. FVEP-P2 may provide Supplementary information on visual pathway or cortical processing, but its contribution beyond established clinical and biomarker-based assessments has not been determined. Despite their distinct primary pathologies, the available studies showed directionally similar FVEP-P2 latency alterations across AD, VaD, and MCI, although the evidence for VaD and MCI remains limited and heterogeneous (4, 35). These findings may reflect convergence on downstream dysfunction in cortical visual-information processing rather than a shared disease mechanism. One possible explanation is that cholinergic degeneration (basal forebrain neuron loss in AD/VaD) may slow visual information transmission (32); similarly, white matter integrity disruption (e.g., subcortical ischemic foci in VaD) (33) could delay cross-regional signal synchronization. It has also been hypothesized that glutamatergic excitability imbalance in early MCI may interfere with phase-locked neural responses. However, these mechanisms have not been directly tested in the context of FVEP-P2 latency and should be regarded as speculative. Notably, PDD also exhibits prolonged P2 latency (210.6 ± 33.7 ms vs. HC: 161.4 ± 34.4 ms; *p* < 0.05). The directionally similar latency alterations observed across AD, VaD, and PDD suggest that FVEP-P2 may reflect non-specific visual pathway or cortical-processing dysfunction rather than a disease-specific electrophysiological signature (25). However, this interpretation remains preliminary because the evidence for VaD and PDD was based on only four comparisons and one comparison, respectively.

Funnel plot asymmetry and Egger’s regression test suggested potential small-study effects in the AD analysis. However, these findings should not be interpreted as definitive evidence of publication bias because the considerable between-study heterogeneity (I^2^ = 88.9%) may itself have contributed to the observed asymmetry. The risk-of-bias assessments also warrant caution. Six of the eight diagnostic-accuracy studies had a high risk of bias in the QUADAS-C patient-selection domain, which may limit the representativeness and external validity of their findings. The NOS assessments identified additional limitations in group comparability and exposure or outcome measurement among some observational studies, indicating possible confounding or measurement error. These concerns support a cautious interpretation of the pooled effect estimates.

Considerable heterogeneity was observed across the principal analyses. Although heterogeneity was lower in the subgroup with a mean age of ≥75 years (I^2^ = 41%), neither the between-subgroup comparison (*p* = 0.56) nor the meta-regression analysis (*p* = 0.491) identified mean age as a statistically significant moderator. These exploratory analyses were based on aggregate study-level means and may have been affected by ecological bias and residual differences in age matching. Other clinical and methodological differences, including diagnostic criteria, disease severity, stimulation frequency (24, 28), electrode configuration and reference location (31), recording equipment, and P2-identification methods, may also have contributed to the remaining heterogeneity. Furthermore, although the pooled estimates were statistically significant, the prediction intervals crossed zero, indicating substantial uncertainty in the effect that might be observed in an individual future study.

Caution is warranted for MCI (7, 23) and VaD (17, 19, 20, 27) analyses due to limited studies (2 and 4, respectively). Small samples increase type II error risk and sensitivity to outliers, while considerable heterogeneity in the VaD analysis (I^2^ = 92.2%) further reduced the certainty of the pooled estimate. Further prospective studies designed to estimate sensitivity, specificity, diagnostic thresholds, and incremental value are required. Before clinical implementation, several issues require resolution. The current evidence does not support FVEP-P2 as a standalone diagnostic marker, because the observed latency prolongation reflects non-specific visual pathway dysfunction rather than a definitive disease signature. Moreover, the presence of similar P2 delays across AD, VaD, and PDD limits its potential value for differential diagnosis. FVEP-P2 should therefore be regarded only as a candidate adjunctive research marker within multimodal assessment frameworks and requires prospective validation using standardized protocols.

This study has several limitations. First, substantial clinical and methodological heterogeneity remained across the principal analyses. The included studies were published between 1983 and 2023, during which dementia diagnostic criteria, FVEP recording systems, signal-processing methods, electrode configurations, stimulus parameters, and P2 peak-identification procedures changed considerably. Variations in disease severity, recruitment settings, age matching, biomarker confirmation, and ophthalmological screening may have further reduced comparability across studies. Although subgroup, meta-regression, and sensitivity analyses were performed, these sources of heterogeneity could not be fully quantified or resolved. Also, although P2 was generally identified as the second positive deflection following flash stimulation, the time windows and peak-identification procedures varied across studies. Consequently, the components labeled as P2 may not have been fully equivalent across all studies, contributing to measurement heterogeneity. Second, the evidence available for MCI, VaD, and PDD was limited. The search strategy was primarily optimized for clinically diagnosed dementia, and only two MCI comparisons, four VaD comparisons, and one PDD comparison were available. Estimates for these conditions were therefore particularly sensitive to individual studies and should be interpreted as exploratory. Third, several studies contributed more than one dementia-subtype comparison using a shared healthy control group. Because individual-participant data were unavailable and the overall analysis could not fully account for the resulting within-study dependence, the pooled estimate across all dementia etiologies may have underestimated statistical uncertainty. Accordingly, the overall dementia estimate was treated as exploratory, with greater emphasis placed on the subtype-specific results. Fourth, the methodological quality of the included studies was variable. Several diagnostic-accuracy studies had a high risk of bias, particularly in patient selection, while some observational studies had limitations in group comparability and outcome assessment. Residual confounding from age, ocular comorbidities, disease severity, and other unmeasured factors could not be excluded, although the direction and statistical significance of the AD estimate were preserved in the restricted sensitivity analysis. The inclusion of different study designs represents an additional limitation. Although all studies provided the same type of patient-versus-control latency data and study design was not a statistically significant moderator in the exploratory meta-regression, differences in participant selection and study conduct may still have affected the pooled estimates. The small and uneven numbers of studies across design categories precluded a reliable design-stratified pooled analysis. Fifth, only English-language full-text publications were included, which may have introduced language bias. In addition, funnel plot asymmetry in the AD analysis suggested possible small-study effects or selective publication, although substantial heterogeneity may also have contributed to this pattern. Finally, the pooled SMDs quantify group-level differences in FVEP-P2 latency but do not establish clinically useful diagnostic thresholds, sensitivity, specificity, or incremental diagnostic value. Prospective studies using standardized diagnostic criteria and FVEP protocols are required before FVEP-P2 can be considered for routine clinical application.

## Conclusion

This review found prolonged FVEP-P2 latency in clinically diagnosed AD, with exploratory findings suggesting similar patterns in MCI and VaD. However, substantial heterogeneity, methodological variability, limited disease specificity, and the small number of studies available for several subtypes preclude the use of FVEP-P2 as a standalone diagnostic or screening tool. FVEP-P2 should currently be regarded as a candidate research marker of non-specific visual pathway dysfunction rather than an established clinical diagnostic marker. Future prospective studies should use standardized diagnostic and electrophysiological protocols and determine whether FVEP-P2 provides incremental value within modern biomarker-supported multimodal assessment frameworks.

## Data Availability

The original contributions presented in the study are included in the article/Supplementary material, further inquiries can be directed to the corresponding author.

## Footnotes

1
https://pubmed.ncbi.nlm.nih.gov/

2
https://www.embase.com/landing?status=grey

3
https://access.clarivate.com/

4
https://www.cochranelibrary.com/
