# Supplementary material for: Flash VEP-P2 latency delay in Alzheimer’s disease and cognitive impairment: a systematic review and meta-analysis
Source: Front Neurol. 2026 Aug 10;17:1772949. doi: 10.3389/fneur.2026.1772949 (PMC13500328; doi:10.3389/fneur.2026.1772949)
Supplement: Supplementary file 1 [file Table_1.DOCX]

**Supplementary Table 1. Summary of Studies Excluded at Full-Text Screening Stage (n = 172)**

| **Exclusion Reason** | **n** |
| --- | --- |
| Review articles or meta-analyses | 85 |
| Studies with fewer than 2 patients (case reports, single-patient studies) | 33 |
| Non-English language publications | 19 |
| Editorials (editorial commentaries, letters to the editor) | 14 |
| No healthy control group (studies comparing only patient subgroups without HC) | 7 |
| Did not report FVEP-P2 latency as a primary outcome (reported P100 latency or VEP amplitude only) | 7 |
| Data presented in figures only; mean ± SD not extractable | 4 |
| NOS score < 5 (critically low methodological quality) | 3 |
| **Total** | **172** |

*Abbreviations: FVEP, flash visual evoked potential; HC, healthy controls; NOS, Newcastle-Ottawa Scale; SD, standard deviation.
Note: Exclusion categories correspond to those reported in the PRISMA flow diagram (Figure 1). Studies excluded at the title/abstract screening stage (n = 431 screened; n = 226 excluded) were not individually listed, as screening at this stage was based on titles and abstracts only.*
