# Supplementary material for: Flash VEP-P2 latency delay in Alzheimer’s disease and cognitive impairment: a systematic review and meta-analysis
Source: Front Neurol. 2026 Aug 10;17:1772949. doi: 10.3389/fneur.2026.1772949 (PMC13500328; doi:10.3389/fneur.2026.1772949)
Supplement: Supplementary file 2 [file Table_2.DOCX]

**Table S2. Detailed Electrophysiological Parameters and Stimulation Conditions of Included Studies**

| **Study** | **Group** | **Sample** | **Age (years)^a^** | **Cognitive Score ^a^** | **P2 Latency (ms)^a^** | **P2 Amplitude (μV) ^a^** | **Stimulation/Recording Parameters** |
| --- | --- | --- | --- | --- | --- | --- | --- |
| Coben-1983 | Mild AD | 40 | 64.2-82.5 ^d^ | CDR 1 | 143.4±18.8 | - | Closed-eye flash; Intensity: Grass PS2 level 2; Electrodes: Bipolar (LO-V, RO-V) |
|  | HC | 40 | Matched | CDR 0 | 139.4±14.9 | - |  |
| Wright-1984 | Early-onset AD (presenile dementia) | 17 | 63.1±6.1 | - | 155.32±24.87 | 17.58±11.09 | Flash: 1363 cd/m², 2 Hz; Electrodes: O1/O2 referenced to Fz |
|  | HC | 17 | 61.8±5.4 | - | 127.41±6.03 | 10.12±9.49 |  |
| Harding-1985 | Early-onset AD (primary presenile dementia) | 20 | 63.7±5.6 | - | 148.4 ± 13.3 | - | Flash: 1363 cd/m², 2 Hz; Electrodes: O1/O2 referenced to C3/C4 |
|  | HC (stratified by age) | 60 | 20-29 ^d^ | - | 114.3 ± 11.45 | - |  |
|  |  |  | 30-39 ^d^ | - | 122.6 ± 11.05 | - |  |
|  |  |  | 40-49 ^d^ | - | 128.8 ± 6.45 | - |  |
|  |  |  | 50-59 ^d^ | - | 125.7 ± 5.15 | - |  |
|  |  |  | 60-69 ^d^ | - | 129.7 ± 7.5 | - |  |
|  |  |  | 70-79 ^d^ | - | 133.0 ± 8.15 | - |  |
| Wright-1986 | Dementia | 41 | 63.8±6.5 | - | 146.9±16.3 | - | Flash: 68 cd/m²/s, 2 Hz; Electrodes: O1/O2  referenced to C3/C4 |
|  | HC | 30 | 64.0±8.3 | - | 129.3±7.1 | - |  |
| Wright & Furlong-1988 | AD | 25 | 80.4±5.3 | - | 157.6±12.6 | - | Flash: Grass PS22 level 2 (68 cd/m²/s), 2 Hz; Electrodes: O1/O2 referenced to C3/C4 |
|  | VaD | 10 | 83.8±4.4 | - | 168.1±25.2 | - |  |
|  | Dementia (total) | 35 | 81.4±5.3 | - | 160.6±26.5 | - |  |
|  | HC | 19 | 79.8±4.7 | - | 137.3±7.3 | - |  |
| Engedal-1989 | AD | 20 | 81.7 ^e^ | 19.8 ^e^ | 158.5 (144-172) ^c^ | - | Flash: Grass stimulator, 2 Hz; Electrodes: 10–20 system. |
|  | VaD | 10 | ≥75 | - | 160.0 (158-172) ^c^ | - |  |
|  | HC | 32 | 79.8±4.7 | 28.8 ^e^ | 130.5(122-135) ^c^ | - |  |
| Philpot-1990 | AD | 25 | 74.5 ^e^ | 15.5±5.1 | 161±29 | - | Flash: 1365 cd/m², 2 Hz; Pattern: 56' checkerboard; Electrodes: O1/O2 referenced to Cz |
|  |  | 12 | ≤74 | - | 137±10 | - |  |
|  |  | 13 | ≥75 | - | 185±48 | - |  |
|  | HC | 13 | 69.9±6.3 | 29.2±0.8 | 128±11 | - |  |
| Brodie-1992 | AD | 7 | 79.2±3.4 | 11±4 | 160.7±8.9 | - | Flash: 40° FOV, 25 cm distance; Electrodes: O1/O2 referenced to Fz |
|  | HC | 7 | 76.9±4.8 | 19±8 | 136.9±7.1 | - |  |
| Sloan-1992 | AD | 40 | 73.8±9.3 | 16.6±5.8 | 146.8±21.6 | - | Flash: Grass PS22 (0.26 lumen·sec/m²); Electrodes: O1/O2 referenced to Fz |
|  | VaD | 32 | 75.2±9.4 | - | 143.3±16.4 | - |  |
|  | HC | 30 | 71.7±5.2 | 28.4±1.6 | 138.8±20.3 | - |  |
| Coburn-1993 | Probable AD | 23 | 75±8 | 17.2±7.4 | 179±19 | - | Flash: 1.5 Mcd, 0.9 Hz; Electrodes: 20-lead 10–20 system; Reference: Average reference |
|  | HC | 11 | 68±5 | 29.4±0.5 | 142±17 | - |  |
| O'Mahony-1993 | PDD | 14 | 78.6±5.3 | 18.1±3.5 | 210.6±33.7 | - | Flash: 0.027 J, 1 Hz; Electrodes: Oz referenced to Fz + mastoid; ERP: Oddball paradigm (target 15%), Fz/Pz referenced to mastoid |
|  | HC | 15 | 77.5±3.8 | 28.9±1.1 | 161.4±34.4 | - |  |
| Haupt-1994 | AD | 15 | 64.6±7.3 | 17.45±6.61 | 121.6±27.2 | 5.3±3.1 | Flash: LED goggles, binocular stimulation; Electrodes: Oz referenced to Fz |
|  | HC | 15 | 63.4±6.2 | - | 124.3±20.6 | 6.0±5.7 |  |
| Sloan-1994 | AD | 27 | 70.1±8.9 | 18.23±5.84 | 158.3±39.5 | 5.5±3.0 | Flash: Grass PS22, intensity 0.26 lumen·sec/m²; Distance: 55 cm; Frequency: 1 Hz |
|  | VaD | 20 | 76.0±8.4 | 20.20±7.08 | 162.9±24.0 | 6.0±4.8 |  |
|  | HC | 15 | 72.7±7.8 | 25.87±3.62 | 142.6±20.3 | 6.0±4.8 |  |
| Grayson-1995 | Probable AD | 10 | 52-74 ^d^ | 23^b^ | 116.22 ± 3.1 | - | Flash: Grass PS22 (level 2), 1 Hz; Reference: Linked ears; Ground: Forehead; Electrodes: 21-lead |
|  | HC | 10 | Matched±2 | 29.3 ^b^ | 109.00 ± 2.3 | - |  |
| Moore-1995 | AD | 31 | 71±9 | 18±4 | 162±32 | - | Flash intensity: 1.5 Mcd (1,500,000 cd); Frequency: 0.9 Hz; Electrodes: 20-lead (10–20 system) |
|  | HC | 31 | 69±5 | 29±1 | 147±20 | - |  |
| Swanwick-1996 | AD | 17 | 71.4±6.9 | 23.9±2.4 | 148.9±4.8 | - | Flash: 0.5 J flash, 2 Hz; Electrodes: O1/O2 referenced to Fz |
|  | HC | 21 | 70.1±6.9 | ≥28 | 128.9±2.6 | - |  |
| Ponomareva-1998 | AD | 20 | 61.9±1.4 | - | 139.3±6.6 | 12.0±1.4 | Flash intensity: 0.3 J; Frequency: Unspecified (analysis duration 500 ms); Electrodes: O1/O2 referenced to C3/C4 |
|  | Aged controls | 20 | 60.8±1.2 | - | 120.1±3.5 | 10.1±0.8 |  |
| Tartaglione-2012 | HC | 11 | 67.9±4.9 | 26.3±1.7 | 140.4±3.1 | - | Flash intensity: 0.5 J; Frequency: 1 Hz; Electrodes: Oz referenced to Cz |
|  | AD-NL | 9 | 70.1±2.7 | 18±2.6 | 137.2±4.6 | - |  |
|  | AD-AL | 10 | 70.7±6.3 | 15.8±3.6 | 153.4±5.1 | - |  |
| Fix-2015 | MCI a | 5 | 78.0±10.3 | 100.50 ± 10.57 | 199.2±13.1 | - | Flash intensity: 5.5 Im·s/ft²; Electrodes: Oz referenced to earlobes |
|  | HC | 8 | 65.6±7.9 | 59.80 ± 9.01 | 159.0±17.6 | - |  |
| Arruda-2023 | AD | 45 | 73.2±8.69 | - | 179.82±19.40 | - | Flash intensity: Unreported ("13.7-cm diameter xenon lamp"); Frequency: Randomized 1.5 ± 0.25 s; Electrodes: Oz |
|  | HC | 60 | 70.7±8.03 | - | 164.20±19.92 | - |  |
| Strigaro-2022 | MCI a | 12 | 73.0±4.7 | 25.4±0.5 | 137.9±12.4 | 11.7±5.0 | Flash intensity: 0.64 J (white light); Electrodes: Oz-Pz reference |
|  | AD | 12 | 72.9±5.1 | 22.3±1.6 | 135.1±16.5 | 13.9±6.5 |  |
|  | HC | 14 | 67.9±4.2 |  | 122.3±16.8 | 12.3±7.3 |  |

AD, Alzheimer's Disease; CDR, Clinical Dementia Rating; HC, Healthy Controls; MMSE, Mini-Mental State Examination; -, Not Reported; PDD, Parkinson's Disease Dementia; RBANS, Repeatable Battery for the Assessment of Neuropsychological Status; SD, Standard Deviation; VaD, Vascular Dementia;

a Mean ± SD；

b Mean

c Median (Interquartile Range)

d Range

e Median

f Cognitive assessment scales used: CDR for Coben-1983; RBANS delayed memory score for Fix-2015; MMSE for all others.

The cognitive scores were all MMSE, unless otherwise specified.

Stimulation parameters: "Flash" refers to flash visual evoked potential (FVEP) unless "Pattern" is specified. Electrode positions follow the International 10–20 system.
