# Supplementary material for: Flash VEP-P2 latency delay in Alzheimer’s disease and cognitive impairment: a systematic review and meta-analysis
Source: Front Neurol. 2026 Aug 10;17:1772949. doi: 10.3389/fneur.2026.1772949 (PMC13500328; doi:10.3389/fneur.2026.1772949)
Supplement: Supplementary file 3 [file Table_3.DOCX]

**Table S3. Diagnostic Criteria and P2 Identification Methods Across Included Studies**

| **Study** | **Dementia Type** | **Diagnostic Criteria** | **P2 Identification Method** | **Notes** |
| --- | --- | --- | --- | --- |
| Brodie-1992 | AD | DSM-III-R + NINCDS-ADRDA | Largest positive peak between 100–300 ms post-stimulus |  |
| Coben-1983 | AD | Clinical diagnosis (pre-NINCDS era); CDR ≥1 | P2 defined as second positive component; latency measured to peak |  |
| Coburn-1993 | AD | NINCDS-ADRDA probable AD | P2 identified as positive peak ~150–200 ms; automated peak detection |  |
| Engedal-1989 | AD + VaD | DSM-III; CT-confirmed VaD | P2 peak latency; visual inspection by two raters | Mixed AD/VaD cohort |
| Fix-2015 | MCI | Petersen MCI criteria | P2 conversion score (Arruda standardized method) |  |
| Grayson-1995 | AD | NINCDS-ADRDA probable AD | P2 peak latency; manual identification |  |
| Harding-1985 | AD | Clinical diagnosis; MMSE <24 | P2 as second major positive deflection; visual inspection |  |
| Haupt-1994 | AD | NINCDS-ADRDA; PET-defined subgroup | P2 latency; peak identified by experienced neurophysiologist | PET-defined subgroup |
| Moore-1995 | AD | NINCDS-ADRDA probable AD; MMSE | P2 peak latency; automated + manual verification |  |
| Philpot-1990 | AD | NINCDS-ADRDA; MMSE | P2 as positive peak 100–250 ms; visual inspection |  |
| Ponomareva-1998 | AD | NINCDS-ADRDA probable AD | P2 latency; peak detection algorithm | Genetic risk subgroup included |
| Sloan-1992 | AD + VaD | DSM-III-R; CT-confirmed VaD | P2 peak latency; visual inspection |  |
| Sloan-1994 | AD + VaD | NINCDS-ADRDA; DSM-III-R VaD | P2 peak latency; visual inspection |  |
| Strigaro-2022 | MCI + AD | Petersen MCI; NIA-AA AD criteria | P2 identified per IFCN guidelines; visual inspection |  |
| Swanwick-1996 | AD | NINCDS-ADRDA probable AD | P2 peak latency; manual identification | Extreme outlier (SMD 5.23) |
| Tartaglione-2012 | AD | NINCDS-ADRDA probable AD; MMSE | P2 latency; automated peak detection |  |
| Wright-1986 | AD | Clinical diagnosis; MMSE | P2 as second positive component; visual inspection |  |
| Wright & Furlong-1988 | AD + VaD | Clinical diagnosis; DSM-III | P2 latency; visual inspection | Mixed AD/VaD cohort |
| Arruda-2023 | AD | Clinical diagnosis | P2 conversion score (standardized Arruda method) |  |
| O'Mahony-1993 | PDD | NINCDS-ADRDA; UK PD Brain Bank criteria | P2 peak latency; visual inspection | Includes Parkinson's dementia |
| Wright-1984 | AD | Clinical diagnosis | P2 latency |  |

*AD, Alzheimer's Disease; VaD, Vascular Dementia; MCI, Mild Cognitive Impairment; PDD, Parkinson's Disease Dementia; NINCDS-ADRDA, National Institute of Neurological and Communicative Disorders and Stroke – Alzheimer's Disease and Related Disorders Association; DSM-III/III-R, Diagnostic and Statistical Manual of Mental Disorders (3rd/revised edition); NIA-AA, National Institute on Aging – Alzheimer's Association; MMSE, Mini-Mental State Examination; CDR, Clinical Dementia Rating; IFCN, International Federation of Clinical Neurophysiology; PET, Positron Emission Tomography; CT, Computed Tomography.*

*Diagnostic criteria and P2-identification methods were extracted from the available study reports. P2-identification procedures varied across studies, including manual visual identification, automated peak-detection algorithms, and combined automated and manual approaches. Information on formal age-matching procedures was not consistently available and was therefore not classified in this table. Group-level age data, when reported, are presented in Supplementary Table S2.*
