# Supplementary material for: Flash VEP-P2 latency delay in Alzheimer’s disease and cognitive impairment: a systematic review and meta-analysis
Source: Front Neurol. 2026 Aug 10;17:1772949. doi: 10.3389/fneur.2026.1772949 (PMC13500328; doi:10.3389/fneur.2026.1772949)
Supplement: Supplementary file 4 [file Table_4.DOCX]

**Table S4. Meta-Regression Results: Moderators of Effect Size (SMD) in Flash VEP P2 Studies**

| **Covariate** | **k (studies)** | **β (SE)** | **95% CI** | **p-value** | **Interpretation** |
| --- | --- | --- | --- | --- | --- |
| Mean age (years) | 18 | 0.02 (0.03) | −0.04 to 0.08 | 0.491 | Older mean age not significantly associated with larger effect size; age-related confounding cannot be excluded |
| Mean MMSE score | 9 | −0.04 (0.05) | −0.14 to 0.06 | 0.415 | Cognitive severity (MMSE) not a significant predictor; limited by small k and restricted MMSE range |
| Publication year | 18 | −0.01 (0.02) | −0.05 to 0.03 | 0.602 | No significant temporal trend; effect sizes have remained relatively stable over the 40-year study period |
| Study design (case-control vs. other designs) | 18 | 0.18 (0.31) | −0.43 to 0.79 | 0.563 | Study design type not a significant moderator; mixed designs do not appear to systematically bias effect estimates |

*SMD, Standardized Mean Difference; SE, Standard Error; CI, Confidence Interval; k, number of studies included in the analysis; MMSE, Mini-Mental State Examination; VEP, Visual Evoked Potential.*

*Meta-regression analyses were performed using univariable random-effects models.*

*β represents the change in pooled SMD per unit increase in the covariate. All analyses should be considered exploratory given the limited number of studies.*
