# Supplementary material for: Flash VEP-P2 latency delay in Alzheimer’s disease and cognitive impairment: a systematic review and meta-analysis
Source: Front Neurol. 2026 Aug 10;17:1772949. doi: 10.3389/fneur.2026.1772949 (PMC13500328; doi:10.3389/fneur.2026.1772949)
Supplement: Supplementary file 5 [file Table_5.DOCX]

**Supplementary materials-Search Strategy**

**1 PubMed**

| **Set** | **Search terms** | **Search type** | **Results** |
| --- | --- | --- | --- |
| #1 | "evoked potentials, visual"[MeSH Terms] OR "evoked potential visual"[Title/Abstract] OR "potentials visual evoked"[Title/Abstract] OR "potential visual evoked"[Title/Abstract] OR "visual evoked potential"[Title/Abstract] OR "visual evoked potentials"[Title/Abstract] OR "visual evoked response"[Title/Abstract] OR "evoked responses visual"[Title/Abstract] OR (("evoke"[All Fields] OR "Evoked"[All Fields] OR "evokes"[All Fields] OR "evoking"[All Fields]) AND "response visual"[Title/Abstract]) OR "responses visual evoked"[Title/Abstract] OR "response visual evoked"[Title/Abstract] OR "visual evoked responses"[Title/Abstract] | Advanced | 21,037 |
| #2 | "Dementia"[MeSH Terms] OR "Dementias"[Title/Abstract] OR "Amentia"[Title/Abstract] OR "Amentias"[Title/Abstract] OR (("Senile"[All Fields] OR "seniles"[All Fields] OR "senility"[All Fields]) AND "paranoid dementia"[Title/Abstract]) OR (("Dementia"[MeSH Terms] OR "Dementia"[All Fields] OR "Dementias"[All Fields] OR "dementia s"[All Fields]) AND "senile paranoid"[Title/Abstract]) OR (("Paranoid"[All Fields] OR "paranoids"[All Fields]) AND "dementia senile"[Title/Abstract]) OR ((("Paranoid"[All Fields] OR "paranoids"[All Fields]) AND ("Dementia"[MeSH Terms] OR "Dementia"[All Fields] OR "Dementias"[All Fields] OR "dementia s"[All Fields])) AND "Senile"[Title/Abstract]) OR ((("Senile"[All Fields] OR "seniles"[All Fields] OR "senility"[All Fields]) AND ("Paranoid"[All Fields] OR "paranoids"[All Fields])) AND "Dementias"[Title/Abstract]) OR "familial dementia"[Title/Abstract] OR "dementia familial"[Title/Abstract] OR "dementias familial"[Title/Abstract] OR "familial dementias"[Title/Abstract] | Advanced | 232,068 |
| #3 | "Neurodegenerative Diseases"[MeSH Terms] OR "neurodegenerative disease"[Title/Abstract] OR "degenerative neurologic disorders"[Title/Abstract] OR "degenerative neurologic disorder"[Title/Abstract] OR (("nervous system diseases"[MeSH Terms] OR ("Nervous"[All Fields] AND "System"[All Fields] AND "Diseases"[All Fields]) OR "nervous system diseases"[All Fields] OR ("Neurologic"[All Fields] AND "Disorder"[All Fields]) OR "neurologic disorder"[All Fields]) AND "Degenerative"[Title/Abstract]) OR (("Neurologic"[All Fields] OR "neurological"[All Fields] OR "neurologically"[All Fields]) AND "disorders degenerative"[Title/Abstract]) OR "nervous system degenerative diseases"[Title/Abstract] OR "neurodegenerative disorders"[Title/Abstract] OR "neurodegenerative disorder"[Title/Abstract] OR (("Degenerative"[All Fields] OR "degeneratively"[All Fields] OR "degeneratives"[All Fields]) AND "diseases nervous system"[Title/Abstract]) OR (("Degenerative"[All Fields] OR "degeneratively"[All Fields] OR "degeneratives"[All Fields]) AND "diseases neurologic"[Title/Abstract]) OR "neurologic degenerative disease"[Title/Abstract] OR (("Neurologic"[All Fields] OR "neurological"[All Fields] OR "neurologically"[All Fields]) AND "degenerative conditions"[Title/Abstract]) OR ((("Degenerative"[All Fields] OR "degeneratively"[All Fields] OR "degeneratives"[All Fields]) AND ("condition s"[All Fields] OR "Conditions"[All Fields] OR "Disease"[MeSH Terms] OR "Disease"[All Fields] OR "Condition"[All Fields])) AND "Neurologic"[Title/Abstract]) OR ((("Degenerative"[All Fields] OR "degeneratively"[All Fields] OR "degeneratives"[All Fields]) AND ("condition s"[All Fields] OR "Conditions"[All Fields] OR "Disease"[MeSH Terms] OR "Disease"[All Fields] OR "Condition"[All Fields])) AND "Neurologic"[Title/Abstract]) OR "neurologic degenerative condition"[Title/Abstract] OR "neurologic degenerative diseases"[Title/Abstract] OR "degenerative neurologic diseases"[Title/Abstract] OR "degenerative neurologic disease"[Title/Abstract] OR "neurologic disease degenerative"[Title/Abstract] OR "neurologic diseases degenerative"[Title/Abstract] OR "degenerative diseases central nervous system"[Title/Abstract] OR "degenerative diseases spinal cord"[Title/Abstract] | Advanced | 451,583 |
| #4 | "Diagnosis"[MeSH Terms] OR "Diagnoses"[Title/Abstract] OR "Diagnose"[Title/Abstract] OR ("Diagnoses"[Title/Abstract] AND "Examinations"[Title/Abstract]) OR ("Diagnoses"[Title/Abstract] AND "Examination"[Title/Abstract]) OR ("Examination"[Title/Abstract] AND "Diagnoses"[Title/Abstract]) OR ("Examinations"[Title/Abstract] AND "Diagnoses"[Title/Abstract]) OR "antemortem diagnosis"[Title/Abstract] OR "antemortem diagnoses"[Title/Abstract] OR "diagnoses antemortem"[Title/Abstract] OR "diagnosis antemortem"[Title/Abstract] OR "postmortem diagnosis"[Title/Abstract] OR "diagnoses postmortem"[Title/Abstract] OR "diagnosis postmortem"[Title/Abstract] OR "postmortem diagnoses"[Title/Abstract] | Advanced | 10,038,289 |
| #5 | #1 AND #2 AND #3 AND #4 | Advanced | 129 |
| #6 | #1 AND #2 AND #4 | Advanced | 175 |
| #7 | #1 AND #2 AND #3 | Advanced | 195 |
| #8 | #1 AND #3 AND #4 | Advanced | 510 |

**2 Embase**

| **Set** | **Search terms** | **Search type** | **Results** |
| --- | --- | --- | --- |
| #1 | 'visual evoked potential'/exp OR 'evoked potentials, visual' OR 'evoked potential visual' OR 'potentials visual evoked' OR 'potential visual evoked' OR 'visual evoked potential' OR 'visual evoked potentials' OR 'visual evoked response' OR 'evoked responses visual' OR 'response visual' OR 'responses visual evoked' OR 'response visual evoked' OR 'visual evoked responses' | Advanced | 29,291 |
| #2 | 'dementia'/exp OR 'dementia' OR 'dementias' OR 'amentia' OR 'amentias' OR 'senile paranoid dementia' OR 'dementias, senile paranoid' OR 'paranoid dementia, senile' OR 'paranoid dementias, senile' OR 'senile paranoid dementias' OR 'familial dementia' OR 'dementia, familial' OR 'dementias, familial' OR 'familial dementias' | Advanced | 575,851 |
| #3 | 'neurodegenerative diseases'/exp OR 'neurodegenerative diseases' OR 'neurodegenerative disease' OR 'degenerative neurologic disorders' OR 'degenerative neurologic disorder' OR 'neurologic disorder, degenerative' OR 'neurologic disorders, degenerative' OR 'nervous system degenerative diseases' OR 'neurodegenerative disorders' OR 'neurodegenerative disorder' OR 'degenerative diseases, nervous system' OR 'degenerative diseases, neurologic' OR 'neurologic degenerative disease' OR 'neurologic degenerative conditions' OR 'degenerative condition, neurologic' OR 'degenerative conditions, neurologic' OR 'neurologic degenerative condition' OR 'neurologic degenerative diseases' OR 'degenerative neurologic diseases' OR 'degenerative neurologic disease' OR 'neurologic disease, degenerative' OR 'neurologic diseases, degenerative' OR 'degenerative diseases, central nervous system' OR 'degenerative diseases, spinal cord' | Advanced | 999,570 |
| #4 | 'diagnosis'/exp OR 'diagnosis' OR 'diagnoses' OR 'diagnose' OR 'diagnoses and examinations' OR 'diagnoses and examination' OR 'examination and diagnoses' OR 'examinations and diagnoses' OR 'antemortem diagnosis' OR 'antemortem diagnoses' OR 'diagnoses, antemortem' OR 'diagnosis, antemortem' OR 'postmortem diagnosis' OR 'diagnoses, postmortem' OR 'diagnosis, postmortem' OR 'postmortem diagnoses' | Advanced | 12,136,770 |
| #5 | #1 AND #2 AND #3 AND #4 | Advanced | 316 |

**3 Web of Science**

| **Set** | **Search terms** | **Search type** | **Results** |
| --- | --- | --- | --- |
| #1 | TS=(Evoked Potentials OR Visual Evoked Potential OR Visual Evoked Potentials OR Visual Evoked Response OR Evoked Responses OR Visual Evoked Responses) | Advanced | 287,593 |
| #2 | TS=(Dementia OR Dementias OR Amentia OR amential OR Senile Paranoid Dementia OR Senile Paranoid OR Paranoid Dementia OR Paranoid Dementias OR Senile Paranoid Dementias OR Familial Dementia OR Familial Dementias) | Advanced | 367,556 |
| #3 | TS=(Neurodegenerative Diseases OR Neurodegenerative Disease OR Degenerative Neurologic Disorders OR Degenerative Neurologic Disorder OR Nervous System Degenerative Diseases OR Neurodegenerative Disorders OR Neurodegenerative Disorder OR Degenerative Diseases OR Neurologic Degenerative Disease OR Neurologic Degenerative Conditions OR Degenerative Condition OR Neurologic Degenerative Condition OR Neurologic Degenerative Diseases OR Degenerative Neurologic Diseases OR Degenerative Neurologic Disease OR Neurologic Disease OR Central Nervous System) | Advanced | 1,202,694 |
| #4 | TS=(Diagnosis OR Diagnoses OR Diagnose OR Examination OR Examinations OR Antemortem Diagnosis OR Antemortem Diagnoses OR Postmortem Diagnosis OR Postmortem Diagnoses) | Advanced | 8,913,265 |
| #5 | #1 AND #2 AND #3 AND #4 | Advanced | 259 |

**4 Cochrane Library**

| **Set** | **Search terms** | **Search type** | **Results** |
| --- | --- | --- | --- |
| #1 | MeSH descriptor: [Evoked Potentials, Visual] explode all trees | Advanced | 576 |
| #2 | (Evoked Potentials, Visual) OR (Evoked Potential, Visual) OR (Potentials, Visual Evoked) OR (Potential, Visual Evoked) OR (Visual Evoked Potential) OR (Visual Evoked Potentials) OR (Visual Evoked Response) OR (Evoked Responses, Visual) OR (Evoked Response, Visual)t OR (Responses, Visual Evoked) OR (Response, Visual Evoked) OR (Visual Evoked Responses) | Advanced | 2,177 |
| #3 | #1 OR #2 | Advanced | 2,177 |
| #4 | MeSH descriptor: [Dementia] explode all trees | Advanced | 10,063 |
| #5 | (Dementias) OR (Amentia) OR (Amentias) OR (Senile Paranoid Dementia) OR (Dementias, Senile Paranoid) OR (Paranoid Dementia, Senile) OR (Paranoid Dementias, Senile) OR (Senile Paranoid Dementias) OR (Familial Dementia) OR (Dementia, Familial) OR (Dementias, Familial) OR (Familial Dementias) | Advanced | 1,013 |
| #6 | #4 OR #5 | Advanced | 10,601 |
| #7 | MeSH descriptor: [Neurodegenerative Diseases] explode all trees | Advanced | 15,644 |
| #8 | (Neurodegenerative Disease) OR (Degenerative Neurologic Disorders) OR (Degenerative Neurologic Disorder) OR (Neurologic Disorder, Degenerative) OR (Neurologic Disorders, Degenerative) OR (Nervous System Degenerative Diseases) OR (Neurodegenerative Disorders) OR (Neurodegenerative Disorder) OR (Degenerative Diseases, Nervous System) OR (Degenerative Diseases, Neurologic) OR (Neurologic Degenerative Disease) OR (Neurologic Degenerative Conditions) OR (Degenerative Condition, Neurologic) OR (Degenerative Conditions, Neurologic) OR (Neurologic Degenerative Condition) OR (Neurologic Degenerative Diseases) OR (Degenerative Neurologic Diseases) OR (Degenerative Neurologic Disease) OR (Neurologic Disease, Degenerative) OR (Neurologic Diseases, Degenerative) OR (Degenerative Diseases, Central Nervous System) OR (Degenerative Diseases, Spinal Cord) | Advanced | 3,188 |
| #9 | #7 OR #8 | Advanced | 17,781 |
| #10 | MeSH descriptor: [Diagnosis] explode all trees | Advanced | 471,775 |
| #11 | (Diagnoses) OR (Diagnose) OR (Diagnoses AND Examinations) OR (Diagnoses AND Examination) OR (Examination AND Diagnoses) OR (Examinations AND Diagnoses) OR (Antemortem Diagnosis) OR (Antemortem Diagnoses) OR (Diagnoses, Antemortem) OR (Diagnosis, Antemortem) OR (Postmortem Diagnosis) OR (Diagnoses, Postmortem) OR (Diagnosis, Postmortem) OR (Postmortem Diagnoses) | Advanced | 225,284 |
| #12 | #10 OR #11 | Advanced | 611,917 |
| #13 | #3 AND #6 AND #9 AND #12 | Advanced | 13 |
